# Supplementary material for: Development and Optimization of a Silica Column-Based Extraction Protocol for Ancient DNA
Source: Genes (Basel). 2022 Apr 13;13(4):687. doi: 10.3390/genes13040687 (PMC9032354; doi:10.3390/genes13040687)
Supplement: Supplementary file 1 [file genes-13-00687-s001.zip › Supplementary Protocol S1.pdf]

EQUIPMENT

1. Incubator capable of agitating the samples
2. Vortex
3. Centrifuge
4. Microcentrifuge
5. MinElute PCR Purification kit (Qiagen, cat no. 28004): contains Qiagen MinElute spin columns, PB binding buffer, PE washing buffer and EB elution buffer.
6. 30K MWCO Vivaspin filters (Sartorius).
7. Eppendorf 1.5 ml LoBind tubes
8. 2 ml (capless) collection tubes
9. 2 ml screw-cap tube (optional)
10. 200 ul long-reach pipette tips

REAGENTS

1. EDTA (0.5M)
2. Urea (1M)
3. Proteinase K (10 ug/uL)
4. USER enzyme, 1,000 units/ml (New England Biolabs)

PROCEDURE

NOTE: this protocol is optimized for approximately 50 mg of bone/tooth powder. Bone powder is preferentially collected in 2 ml screw cap tubes that enable them to be rotated during incubation.

Reagent preparation (hands-on time: less than 15 minutes per batch)

1. For each sample, prepare an extraction buffer mix as follows:
   i. 630 uL EDTA (0.5M, pH 8)
   ii. 70 uL UREA (1M)

Demineralization and DNA digestion (hands-on time: 30-60 minutes per batch)

2. Add 700 uL of extraction buffer mix to each sample separately, and vortex thoroughly.

3. Add 15 uL of Proteinase K (10 ug/uL) to each sample separately, and vortex thoroughly.

4. Incubate the tubes under motion overnight (12-24h) at 55°C.

DNA purification (hands-on time: 1-2 hours per batch)

5. Pellet the bone powder by centrifugation for 5 minutes at 2,300 rpm.

6. Transfer the supernatant to a Vivaspin filter.

NOTE: Avoid disturbing the bone pellet, as small bone particles can clog up the filter. It is advised to not transfer more than 700 uL of supernatant.

NOTE: Avoid spilling liquid on the rim of the Vivaspin filter. This may create a vacuum that results in the supernatant not flowing through.

7. Centrifuge at 12,000 rpm until less than 120 uL supernatant is left
   The flow-through times depend on the sample but will often range between 7 to 10 minutes. If more than 120 uL is left after 10 minutes, it is advised to repeat the centrifuge step for 5 minutes, until enough liquid has flown through.

   NOTE: The opening of the Vivaspin filter lids should face the center of the centrifuge. This is essential to make sure that the centrifugal force works towards the filter.

   NOTE: Do not centrifuge at higher speeds, as this can break the filter.

8. Add 600 uL (or ≥ 5X) of PB binding buffer to 1.5 mL a LoBind tube

9. Transfer the concentrated liquid in the Vivaspin filter, using long-reach tips, to the LoBind tube with PB binding buffer and vortex thoroughly. Subsequently spin the mixture down.
   NOTE: Avoid touching the filter of the Vivaspin column while pipetting the liquid.

10. Transfer the sample + PB buffer mixture (max 720 uL) to a MinElute spin column and centrifuge for 1 minute at 13,000 rpm.

11. Discard the flow-through and transfer the column to a new collection tube.
    NOTE: PB buffer contains a high concentration of guanidine hydrochloride and should thus be handled as hazardous waste.

12. Apply 720 uL of PE washing buffer onto the column and centrifuge for 1 minute at 13,000 rpm.

13. Discard the flow-through and transfer the column to a new collection tube.

14. Centrifuge the column for 1 minute at 13,000 rpm.
    This step is to make sure that any residual ethanol is removed from the MinElute column.

15. Discard the flow-through and transfer the column to a 1.5 mL LoBind Eppendorf tube
    i. Cut of the lids of the LoBind tube
    ii. Leave the column to dry for ~5 minutes with open lid
    This step is added to make sure that any residual ethanol evaporates from the

MinElute filter.

16. Add 50 uL of EB Buffer to the column and leave for 1 minute.
    NOTE: The EB buffer can also be warmed to 37°C to increase elution efficiency.

17. Centrifuge the column for 1 minute at 13,000 rpm.

18. Add another 50 uL of EB Buffer and leave for 1 minute.

19. Centrifuge the column for 1 minute at 13,000 rpm.

20. Transfer the DNA extract from the lid-less tube to a new 1.5 mL screw cap Eppendorf tube or LoBind tube.

Uracil treatment

21. Incubate 20 uL ancient DNA extract with 3 uL of USER enzyme for 3 hours at 37°C. Stop the reaction by cooling the mixture to 4°C.

Alternatively, if sequencing libraries are made following the protocol of Meyer and Kircher (2010), one can combine uracil treatment with the blunt-end repair step before adding T4 DNA polymerase.
