# Supplementary figures and images for: Development and Optimization of a Silica Column-Based Extraction Protocol for Ancient DNA
Source: Genes (Basel). 2022 Apr 13;13(4):687. doi: 10.3390/genes13040687 (PMC9032354; doi:10.3390/genes13040687)

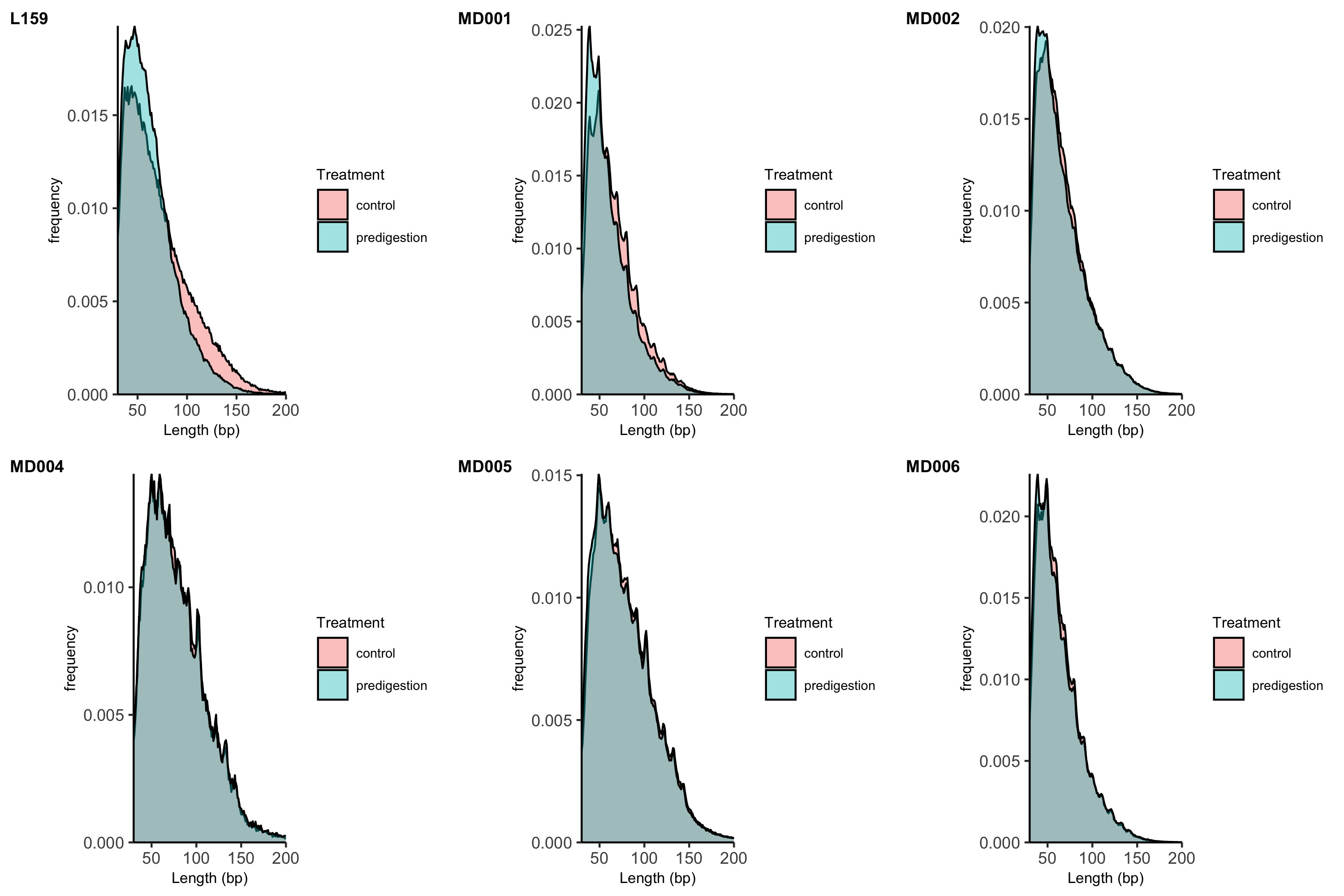

Supplement: Supplementary file 1 [file genes-13-00687-s001.zip › Figure S1.jpeg]

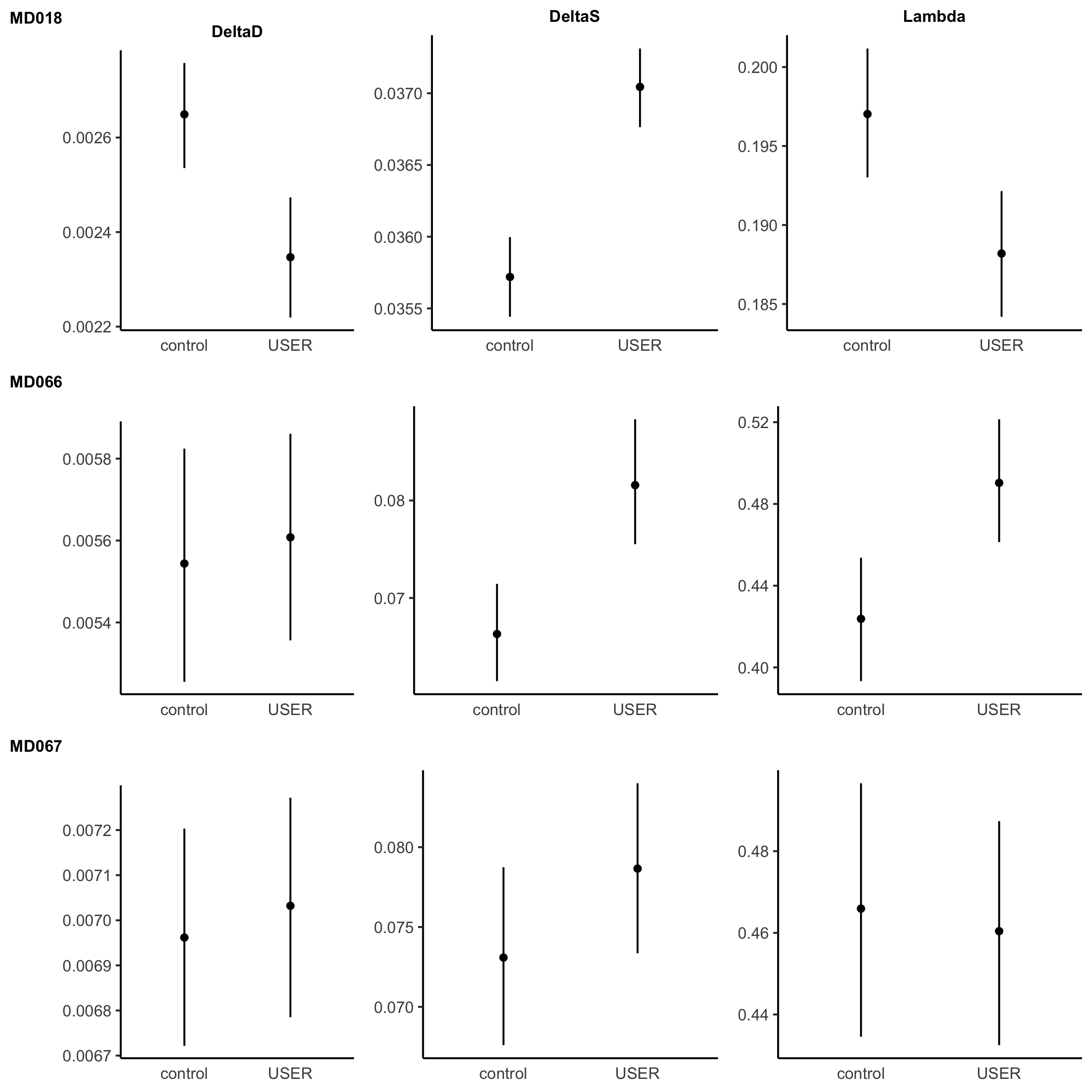

Supplement: Supplementary file 1 [file genes-13-00687-s001.zip › Figure S10.jpeg]

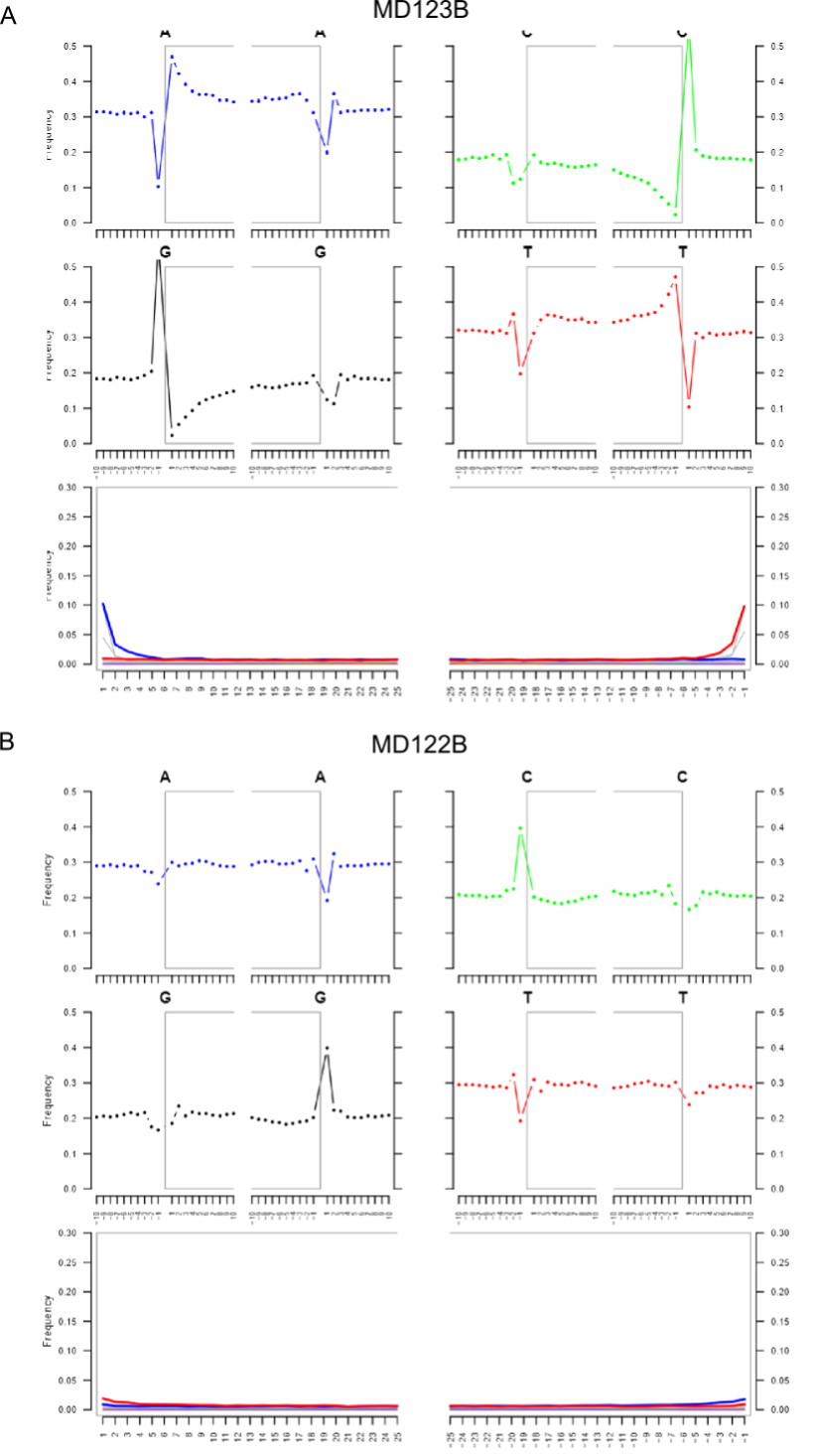

Supplement: Supplementary file 1 [file genes-13-00687-s001.zip › Figure S11.jpeg]

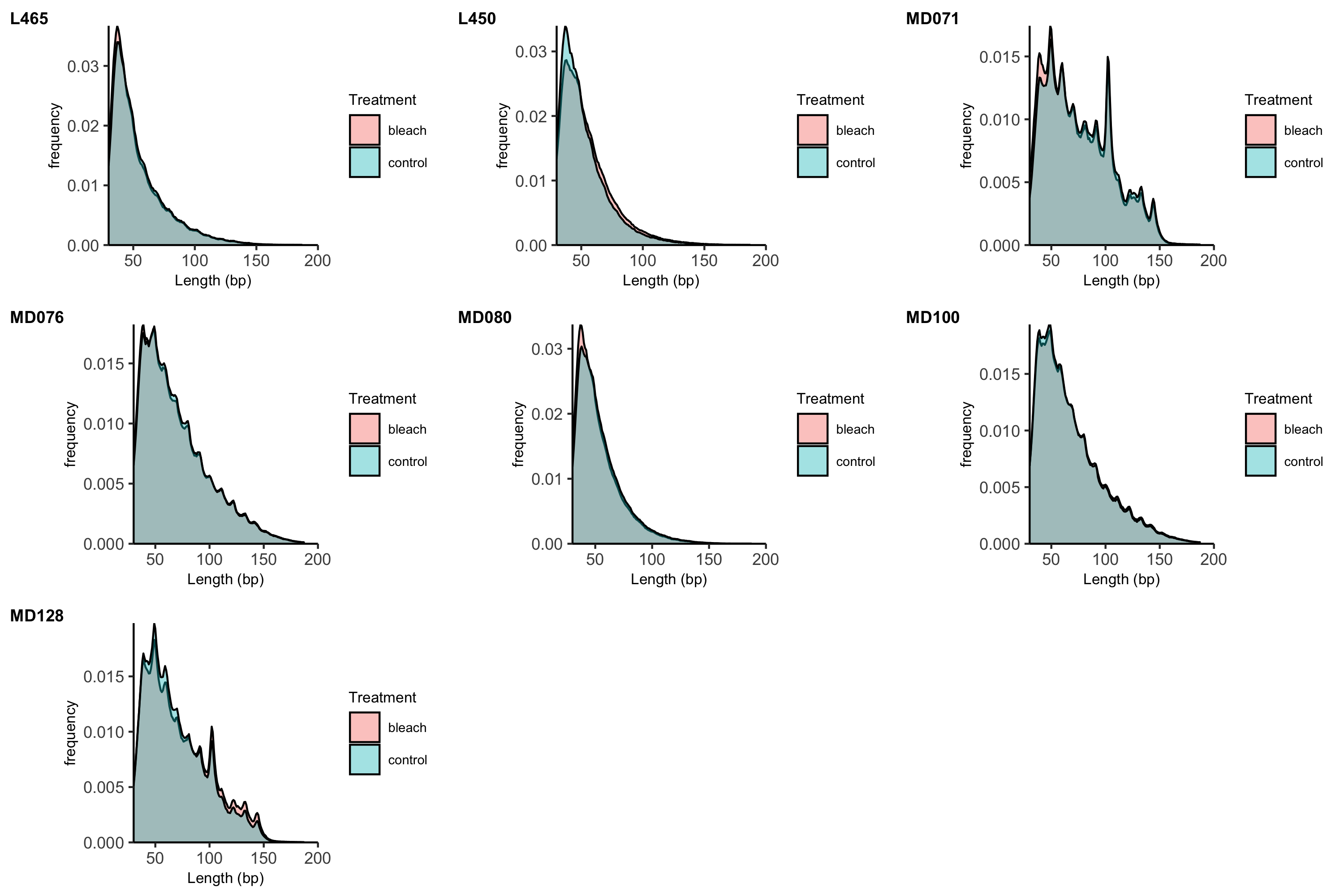

Supplement: Supplementary file 1 [file genes-13-00687-s001.zip › Figure S2.jpeg]

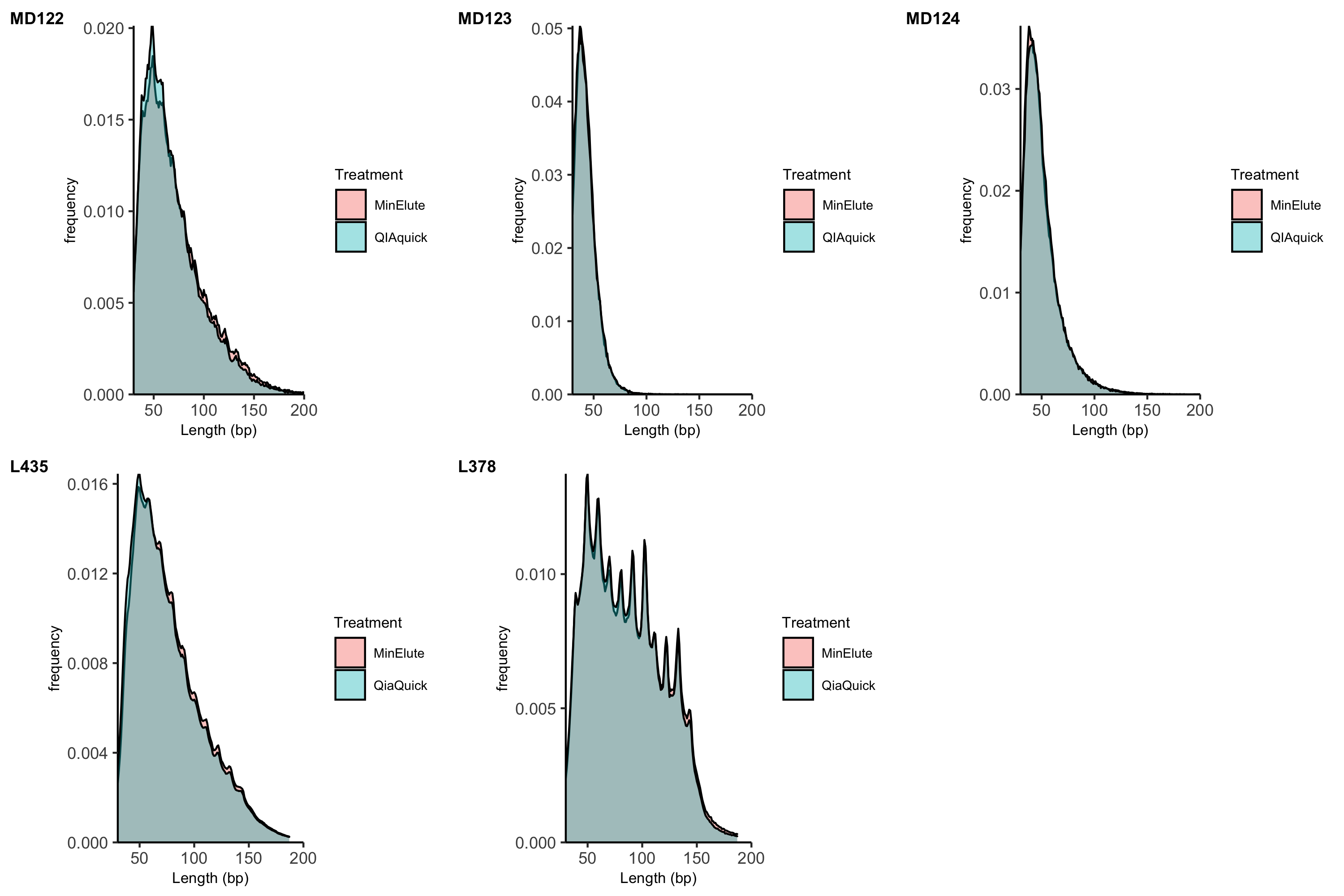

Supplement: Supplementary file 1 [file genes-13-00687-s001.zip › Figure S3.jpeg]

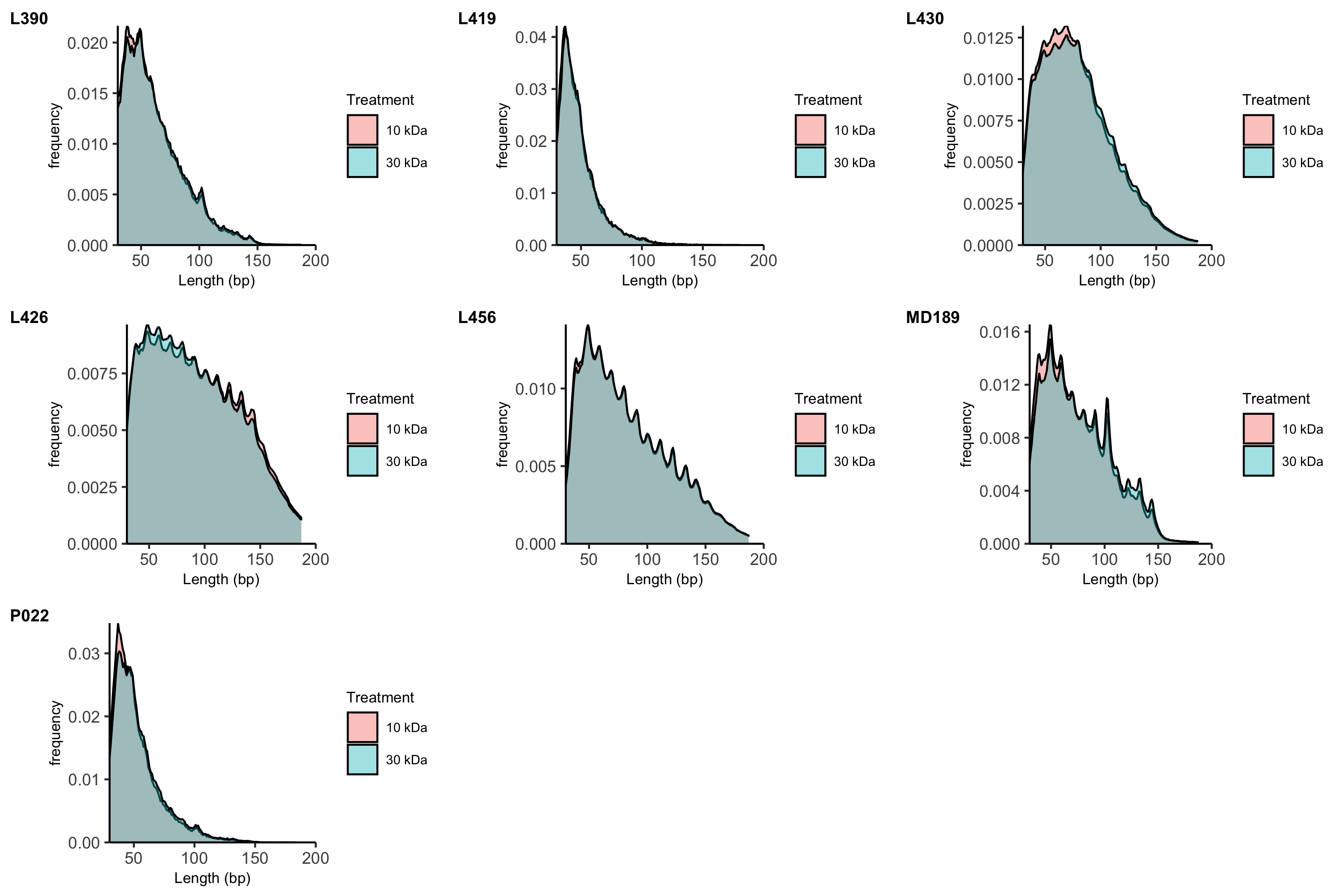

Supplement: Supplementary file 1 [file genes-13-00687-s001.zip › Figure S4.jpeg]

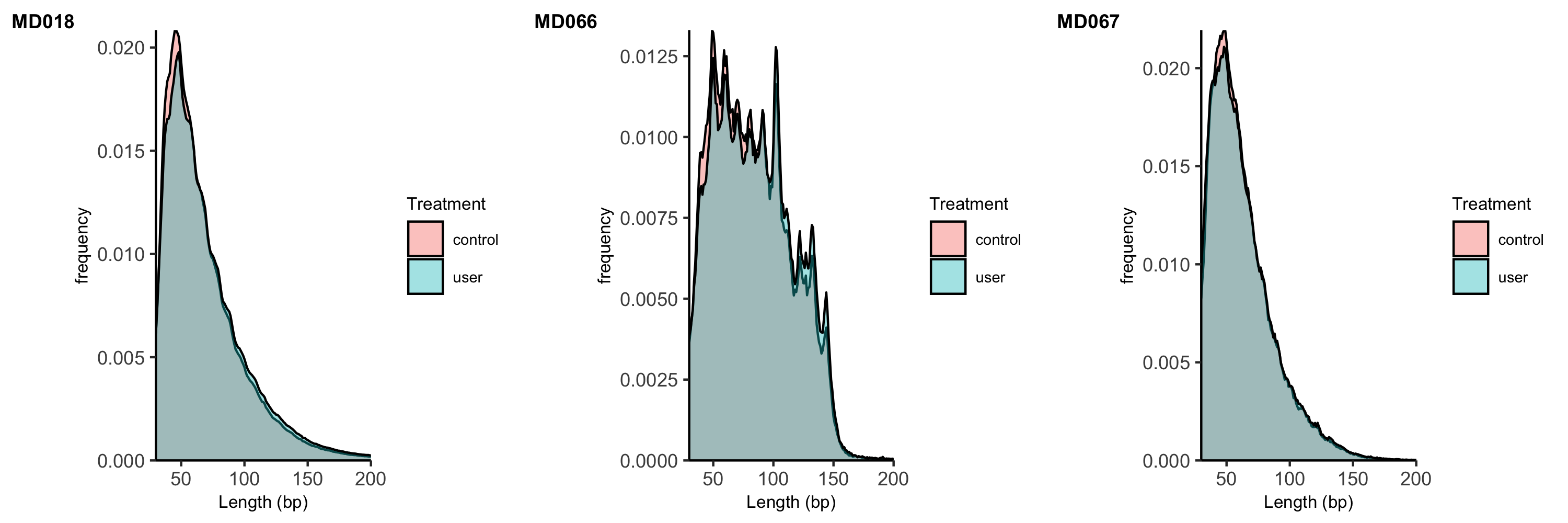

Supplement: Supplementary file 1 [file genes-13-00687-s001.zip › Figure S5.jpeg]

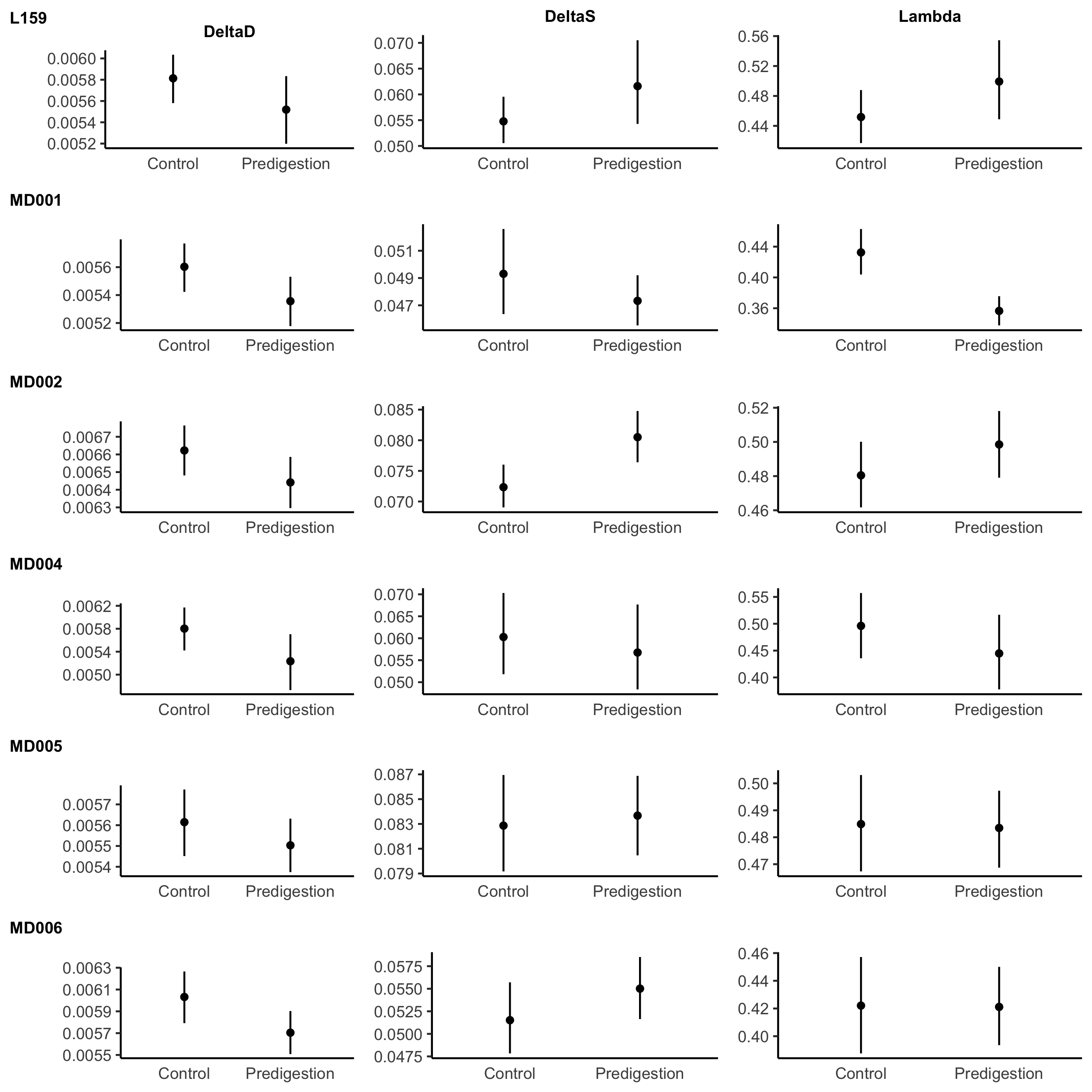

Supplement: Supplementary file 1 [file genes-13-00687-s001.zip › Figure S6.jpeg]

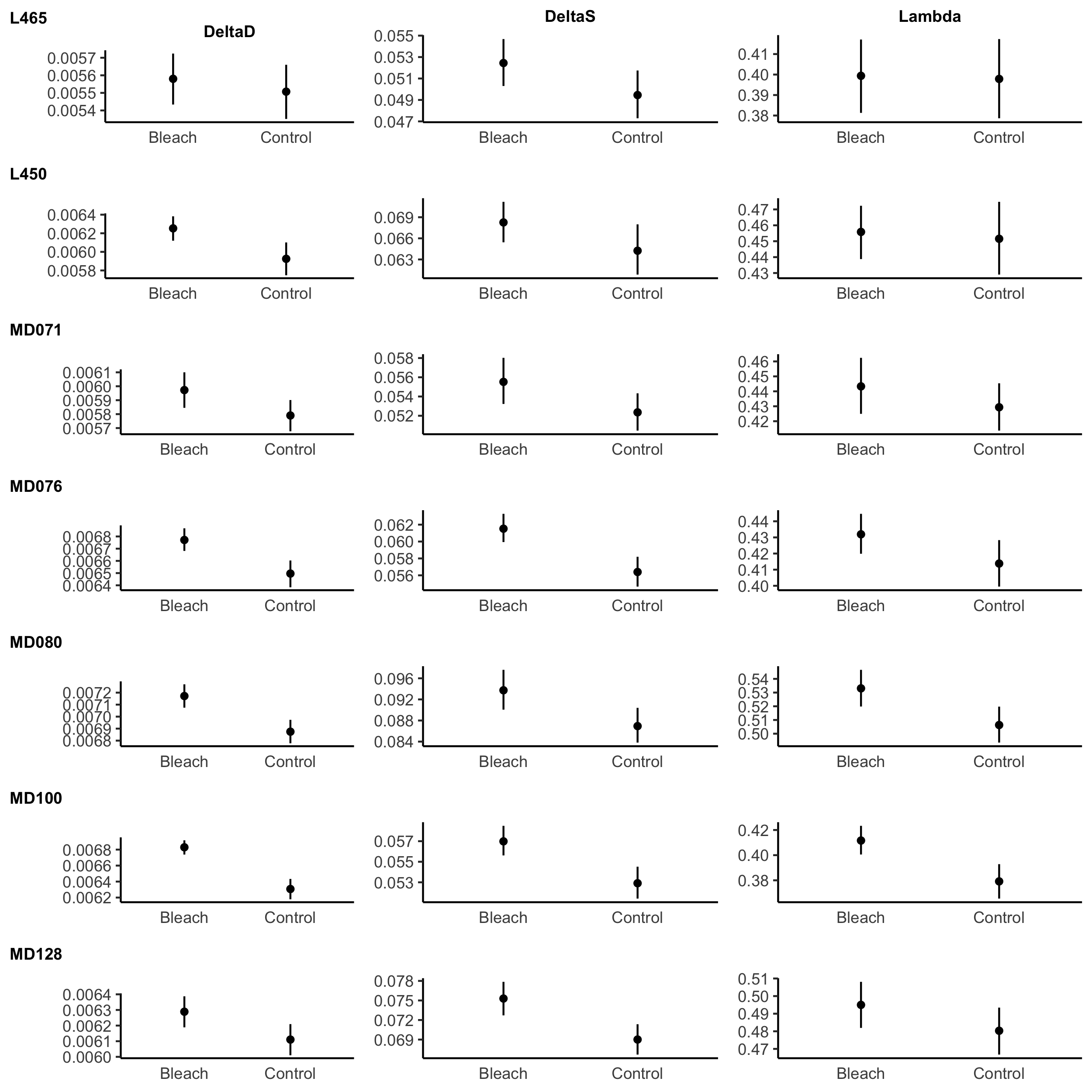

Supplement: Supplementary file 1 [file genes-13-00687-s001.zip › Figure S7.jpeg]

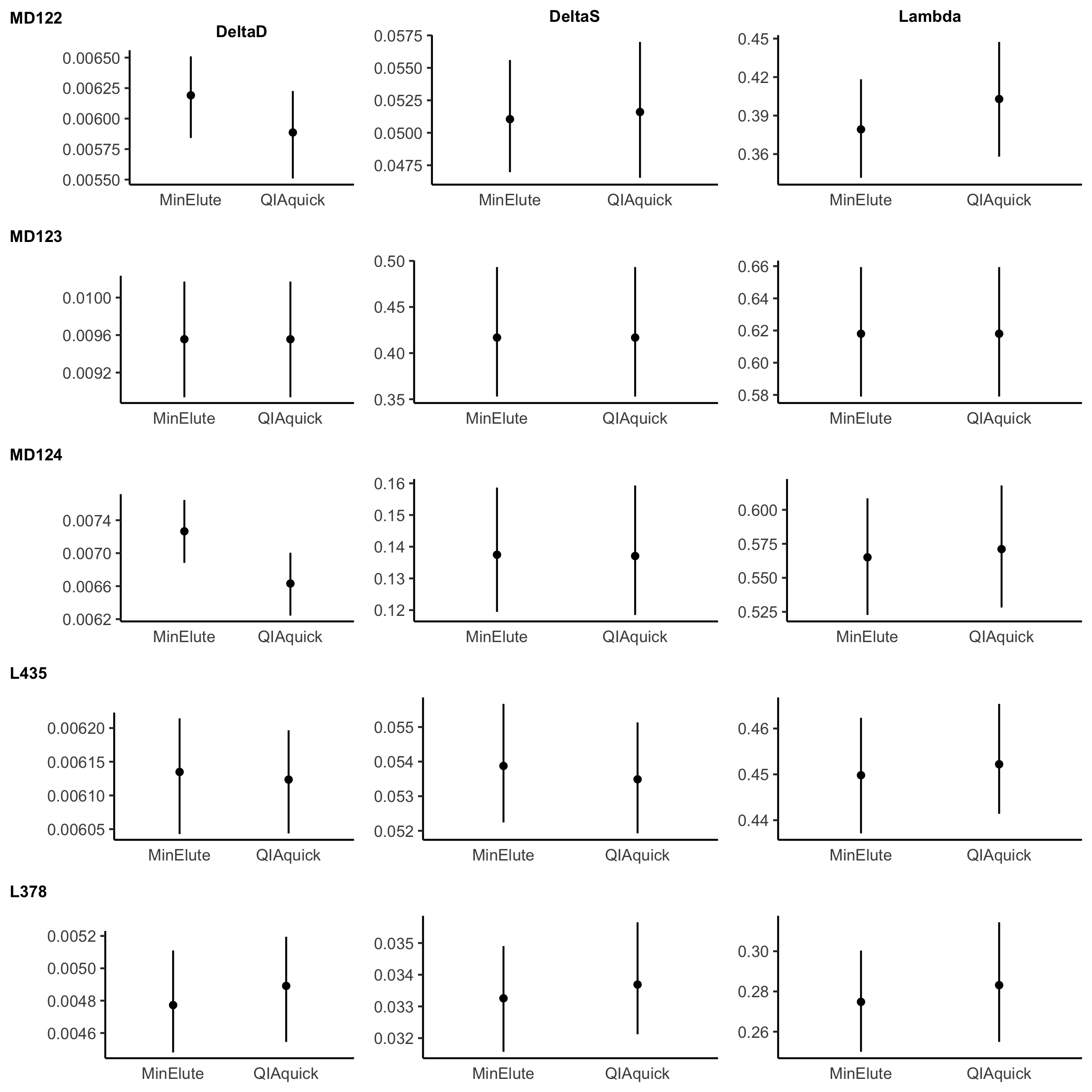

Supplement: Supplementary file 1 [file genes-13-00687-s001.zip › Figure S8.jpeg]

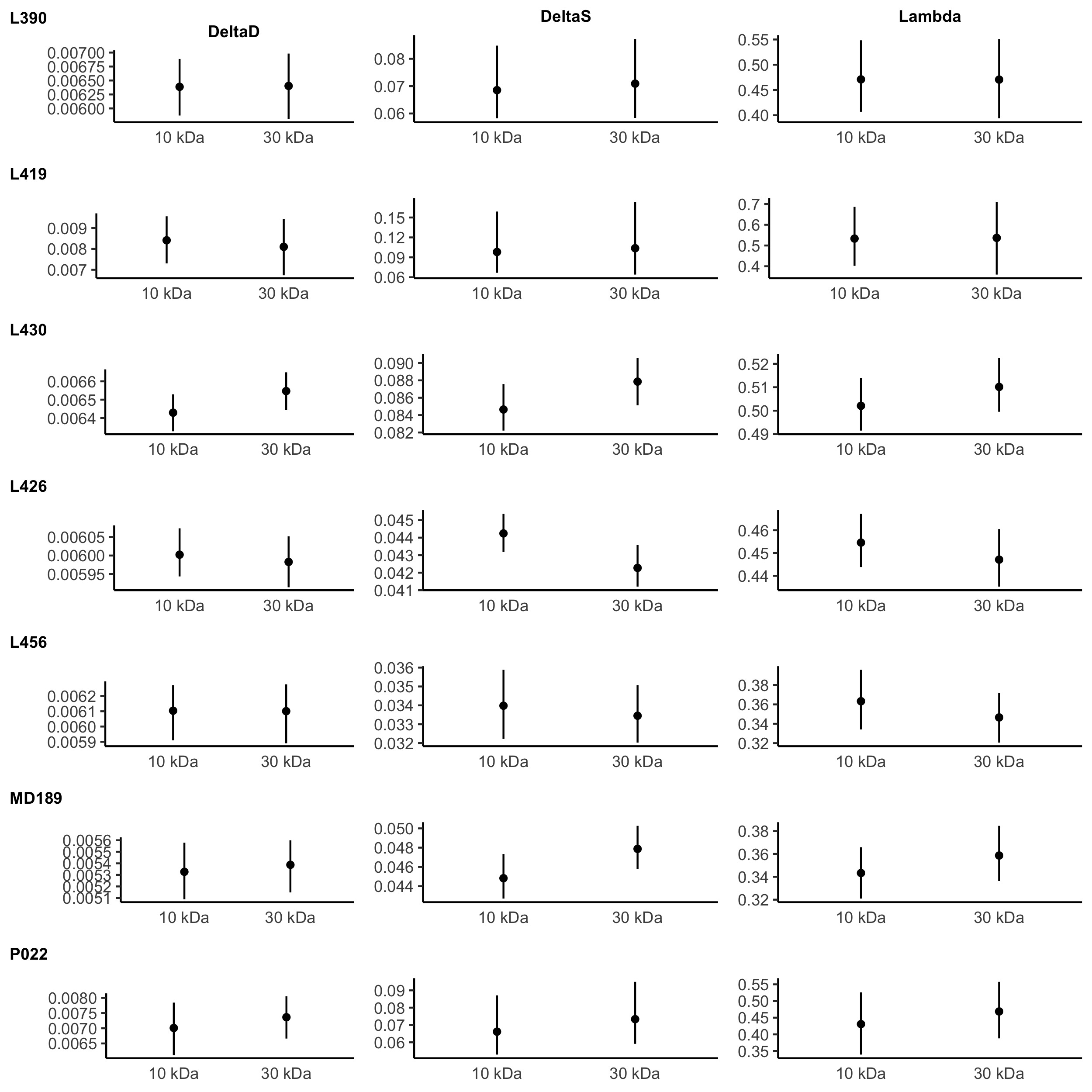

Supplement: Supplementary file 1 [file genes-13-00687-s001.zip › Figure S9.jpeg]
